# Nonkeratinised Squamous Metaplasia of the Urinary Bladder in Children: A Report of Case Experiences

**DOI:** 10.1155/2014/936970

**Published:** 2014-04-15

**Authors:** Beata Jurkiewicz, Tomasz Ząbkowski

**Affiliations:** ^1^Pediatric Surgery Department, Children's Hospital, Marii Konopnickiej Street 65, 05-092 Dziekanów Leśny, Poland; ^2^Urology Department, Military Medical Institute, Szaserów Street 128, 04-349 Warsaw, Poland

## Abstract

*Background*. Squamous metaplasia refers to the pathological transformation of the urothelium leading to nonkeratinised stratified squamous metaplasia (N-KSM). *Objective.* To present our experiences in the diagnosis and treatment of N-KSM of the urinary bladder in children. *Materials and Methods*. In this study, we present our experiences in the diagnosis and treatment of N-KSM of the urinary bladder in children aged from 5 to 17 years. From 2005 to 2013, metaplasia was diagnosed in 119 patients. The reasons behind visiting the hospital were nonspecific intense pain in the abdomen, recurrent urinary tract infections, and urination disorders. The most common symptoms of urinary bladder dysfunction were pollakiuria and difficulties in initiating micturition and retention of urine (reduced detrusor muscle activity). *Results*. In 20/119 patients (16.8%), metaplasia was incidentally diagnosed during cystoscopy performed for other causes. The changes characteristic for squamous metaplasia were diagnosed—in all these patients, a biopsy was performed. In all 119 patients, a squamous metaplasia was histopathologically diagnosed. *Conclusions.* Squamous metaplasia of the urinary bladder mucosa occurs in children and adolescents. Symptomatic treatment is administered mainly to improve the patients' quality of life and disease prognosis.

## 1. Introduction

**Figure 1:**
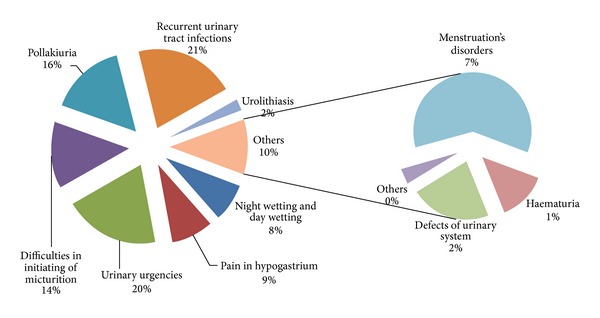
Rate of the most frequent metaplasia symptoms.

Metaplasia is defined as the transformation of the urothelium leading to nonkeratinised stratified squamous metaplasia (N-KSM) and is a well-known gynecological disorder in adult, usually menopausal, women [1, 2] (Figure 1). In the literature, there are very few reports of this disease in maturing girls [3–5]. In 2002, Ankem et al. presented a case of urinary bladder metaplasia, as an incidental finding, in a 15-year-old girl. They only recommended further observation and gave no suggestions for further treatment [6].

The dysuric disorders with concomitant intense pain in the hypogastrium, in females and males, are caused by various disorders in the urinogenital system as follows:

- painful bladder syndrome: pain and discomfort above the pubic area with concomitant pollakiuria,
- urethral pain syndrome: recurrent episodes of pain, especially during micturition with concomitant pollakiuria,
- vulval pain syndrome,
- vaginal pain syndrome,
- scrotal pain syndrome,
- perineal pain syndrome,
- pelvic pain syndrome.

The causes of pain in squamous metaplasia of the bladder are unknown. The pain is usually attributed to the disruption of the layer of glycosaminoglycans (GAGs, compounds of composite structure, having a negative charge) covering the urothelium. This disruption leads to the “syndrome of leaking epithelium” that allows penetration of allergens, chemical irritants, medicines, toxins, and potassium ions into the bladder tissue, consequently resulting in painful bladder syndrome [7].

Some studies have reported the occurrence of squamous metaplasia in neurogenic bladder, especially among those undergoing bladder emptying using an indwelling catheter [8, 9]. Studies on the vanilloid receptor TRPV1 in the submucosa have explained the underlying mechanism of detrusor sphincter dyssynergia. Excessive actuation of the receptor leads to pain and pollakiuria, followed by full anergy. Fluctuations in the pH of the bladder environment cause irritation of the receptors and resultant discomfort and bladder disorders [10, 11]. The presence of muscarinic and nicotinic receptors in the urothelium cells might indicate the active participation of the urothelium in the regulation of bladder function. Acetylcholine, released under the influence of different impulses (mechanical, biochemical, etc.), stimulates the nervous system, leading to excessive action of the detrusor muscle and consequent pollakiuria [12]. Some studies on adult urology have discussed treating painful bladder syndrome by using bladder instillation of hyaluronic acid (HA), as the intravesical use of HA might help reconstruct the damaged layer of GAGs on the urothelium; unfortunately, the results of these studies are poorly documented [13, 14].

## 2. Materials and Methods

**Figure 2:**
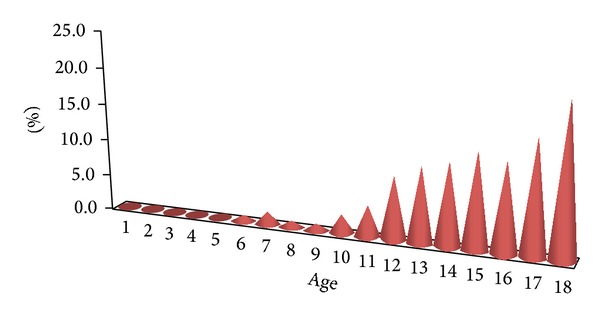
Number of metaplasia diagnosis depending on age of child.

From 2005 to 2013, 1570 cystoscopies were performed in the Pediatric Surgery Department. The main reasons behind visiting the hospital were recurrent urinary tract infection, urinary urgencies, pollakiuria, difficulty in initiating micturition, pain in hypogastrium, night wetting and day wetting, menstruation's disorders, urolithiasis, defects of urinary system, and hematuria (Figure 1). Among this group of patients, there were 711 (45.3%) boys and 859 (54.7%) girls aged between 1 month and 18 years. The changes in bladder mucosa macroscopically visible in cystoscopy were diagnosed in 415 (26.4%) patients. In 202 (12.8%) children, cystitis cystica was diagnosed, in 94 (5.9%) cases, cystitis chronica was diagnosed, and in 119 (7.5%) children, the changes characteristic for squamous metaplasia were diagnosed; in all these patients, a biopsy was performed. In all 119 patients, a squamous metaplasia was histopathologically diagnosed. Thereby, a study group consisted of 119 children (116 girls and 3 boys) and aged between 5 and 17 years (mean age, 13.7 years) (Figure 2).

**Figure 3:**
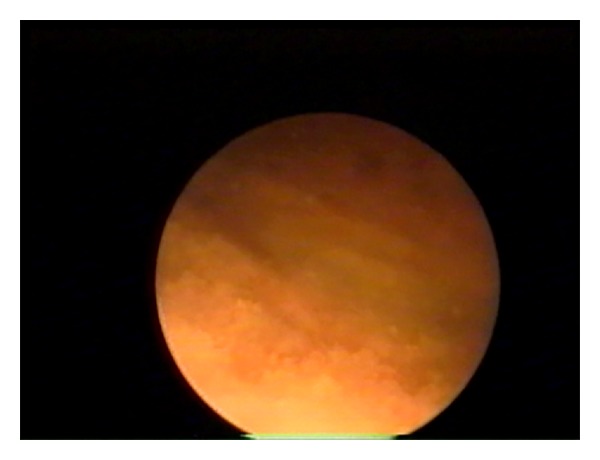
Cystoscopy—an image of squamous metaplasia.

**Figure 4:**
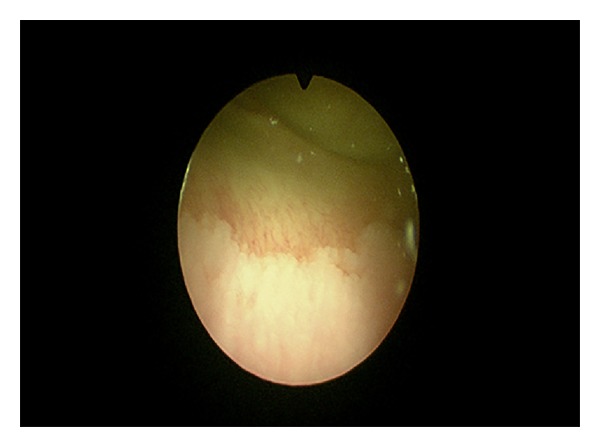
Cystoscopy—an image of squamous metaplasia.

The follow-up duration was 1–8 years. In 20/119 patients (16.8%), metaplasia was incidentally diagnosed during diagnostic cystoscopy for treating urolithiasis or congenital defects of the lower urinary tract (Figures 3 and 4).

**Figure 5:**
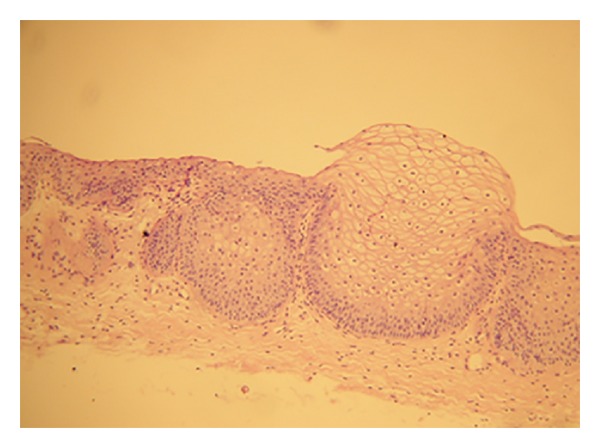
Histopathology examination—a nest of nonkeratinising stratified squamous epithelium; no keroid layer; basally connective tissue.

**Figure 6:**
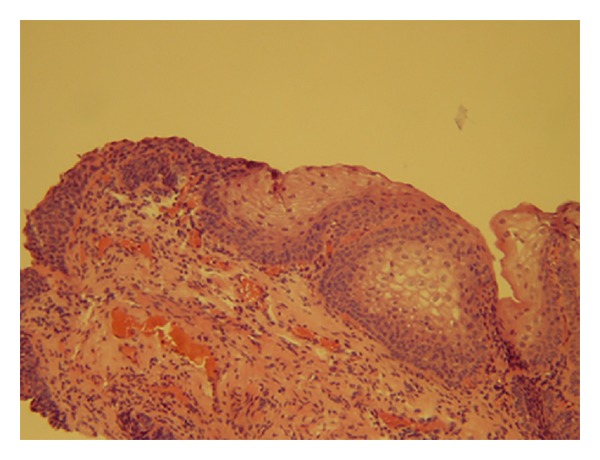
Histopathology examination—nonkeratinising stratified squamous epithelium.

The disruption of the urothelium was usually found at the neck and triangle of the bladder and was rarely seen beyond the interureteric fold (Figures 5 and 6).

On immunohistochemical examination of the specimens, macroscopic changes were observed. We aimed to exclude or confirm the probability of hyperplastic development in the bladder on the basis of the following:

- low level of protein p53 (“the guardian of genome integrity”),
- presence of caspase enzyme, indicating that an appropriate number of cells are going through apoptosis,
- presence of cytokeratins AE-1/AE-2, indicating the presence of stratified squamous epithelium,
- decreased antigen ki-67 staining on the perimeter, indicating that the epithelium is unable to proliferate,
- high level of glycogens in the epithelial cells, indicating the presence of infection, for example, infection with HPV or excess of anabolic hormones—infection with HPV was not confirmed on other tests.

All the children who showed detrusor overactivity and decreased bladder volume underwent urodynamic examination. In 80% of the girls aged over 16 years, difficulties in initiation of detrusor contraction in the micturition phase, which usually brings about adequate susceptibility of bladder wall and proper urine flow, were seen, without features of bladder outlet obstruction. After micturition, no urine residue was found in the bladder.

Owing to the absence of an established treatment modality for squamous metaplasia of the urinary bladder in children, we developed our own treatment modalities. Children presenting with recurrent urinary tract infections on medical interview were subjected to ultrasonography of the urinary system, repeated urinalysis, and urine culture tests. Then, on the basis of antibiogram findings, antibiotic and chemotherapeutic treatment was administered to eliminate the bacteriological factors. Second-generation cephalosporin was prescribed for 10 days and then treatment crossover with chemotherapeutics in therapeutic dose (change in every week) during 3 months. After 3 months, nitrofurantoin was recommended at maintenance doses. However, for those with serious infections and cystitis cystica changes (15 cases [12.6%]), cystoclysis was additionally performed with 1% aminoglycoside solution according to the following protocol: 2 times per day for 10 days, followed by once daily for 1 month.

Furthermore, the children were administered Uro-Vaxom, 1 tablet for 3 months; owing to the immunomodulatory activity of Uro-Vaxom, we expected a favorable effect on the regeneration of the bladder mucosa. Uro-Vaxom is generally prescribed in cases of stimulation of natural killer cells, activation of B lymphocytes, increased immunoglobulin IgA secretion, increased interleukin-1 secretion, stimulation of T-lymphocyte activity, or increase in the alveolar volume of macrophages [12]. Vitamin A was prescribed at a dose of 1 tablet twice daily to eliminate the likelihood of vitamin A deficiency that could influence the regeneration of the bladder mucosa.

Disorders of bladder activity with regard to detrusor overactivity, decreased micturition volume, increased sensitivity in the bladder, right susceptibility of bladder wall, and right outline of flow curve were observed in 94 patients, that is, 77% of all children with metaplasia. In case of detrusor muscle overactivity, oxybutynin or solifenacin succinate was administered long-term until subjective well-being was observed on urodynamic examination. The 36 (29.5%) girls who showed no response to pharmacological treatment were prescribed additional exercises and biofeedback therapy for improving the functioning of the diaphragm and pelvic muscles and promoting full emptying of the urinary bladder.

In 27 girls, it was diagnosed menstruation's disorders. In menstruating girls, the levels of estrogen and progesterone were determined in the luteal phase (27 examinations were performed).

Further, the children diagnosed with squamous metaplasia were observed and treated for 1 year, followed by cystoscopy and biopsy of the bladder to obtain specimens for histopathological examination. On cystoscopy, the presence of abnormal tissues and morphological changes in the stratified squamous epithelium of the bladder were observed. Thereafter, urodynamic examinations were performed to determine disruptions in bladder activity.

## 3. Results

Control group examinations performed after one year showed no progression of the condition on cystoscopic images. There was no transformation of urothelial epithelium of urinary bladder into nonkeratinised stratified squamous epithelium. In 23 (18.8%) of the cases, the area of metaplasia was determined to be relatively larger when compared to the previous examination.

**Figure 7:**
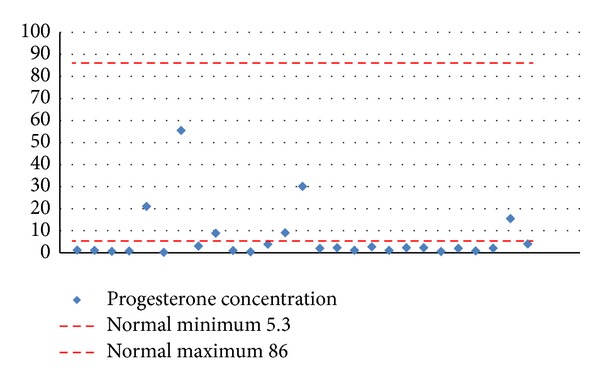
Progesterone concentration in blood serum in girls with squamous metaplasia.

The level of progesterone in menstruating girls was lower than the reference range in 21 (78%) girls (Figure 7).

In all, 99 patients (96 girls and 3 boys) were pharmacologically treated (with oxybutynin or solifenacin succinate) for micturition disorders, and 74 (60.6%) patients showed subjective improvement in bladder activity after treatment. The improvement in bladder function could be attributed to the introduction of exercises involving the bladder muscles, diaphragm, and pelvis and the biofeedback therapy. In subsequent urodynamic examinations, increase in bladder volume, decrease in detrusor overactivity, and increase in intervals between micturition were observed. However, detrusor overactivity recurred periodically after weaning of oxybutynin. In 20 (16.3%) children, no progression in bladder dysfunction was observed, so pharmacological treatment and biofeedback therapy were continued. Dysuric symptoms were still evident, such as pain on micturition, pollakiuria, feeling of bladder fullness, and difficulties in initiating micturition. These disorders also influenced the general development of these children. Their academic performance was unsatisfactory, and their ability to concentrate and work in groups deteriorated. In the cases of 11 girls (9%; aged 15–17 years), complaints of pain prevented their normal participation in the lessons at school. These girls were referred for individual teaching and psychotherapy. In case of 20 girls in whom metaplastic changes in the bladder were diagnosed incidentally, antibacterial treatment was not prescribed.

During continuous antibacterial treatment in 1 year, there was no recurrence of infection in 75 (61.4%) patients. In 24 patients (20.1%), recurrence of urinary tract infection was observed, despite continuous, chronic treatment. In urine culture tests, findings of Gram-negative rods were most prevalent,* Escherichia coli*,* Klebsiella pneumoniae*,* Pseudomonas aeruginosa*,* Proteus mirabilis*,* Citrobacter*,* Enterobacter*, and Gram-positive rods of* Staphylococcus epidermidis*. In 4 cases, mycotic infection caused by* Candida albicans* was observed. The children with recurrent infection were treated according to the antibiogram findings.

All the children were recommended a special diet so that they did not consume bladder-irritating substances. They were recommended to exclude the following from their diet: citrus fruits, pepper, chili, strong spices, cranberry, vitamin C, and products causing urine acidification.

## 4. Discussion

N-KSM of the urinary bladder is known to occur mainly in women aged above 50 years [1, 2, 15]. The recommended management strategy involves periodic routine cystoscopy for early detection of the transformation of stratified epithelium into neoplastic cells. Many authors have recommended symptomatic treatment for this disorder [16–18].

The causes of metaplasia in women are as follows.

Systemic:

- vitamin A deficiency,
- genetic disorder involving the secretory glands.

Local:

- chronic inflammation,
- hormonal imbalance, for example, estrogen and progesterone,
- infection with Schistosoma (*Schistosoma haematobium*),
- recurrent urinary tract infections,
- surgical procedures performed on the urinary bladder,
- neurogenic bladder disorder.

Metaplasia might be one of the causes of painful bladder syndrome. Chronic pain in the hypogastrium also influences the functioning of these patients in society. The management of these painful syndromes is changing. In the latest guidelines for treating chronic pelvis pain, the recommended treatment strategies are as follows: treatment of infection with antibiotics, bladder instillation with HA or BCG, and psychological therapy [19]. Only single authors of publications refer to changes in bladder and generating symptoms which might comply with painful bladder syndrome in maturing children [4, 6, 20–23].

The girls with metaplastic changes in the bladder had dysfunctional relations with their peers. The pain, pollakiuria, and urinary urgencies hindered their progress in school. In the cases of 11% of the girls, the pain had an impact on their learning. These girls were recommended to visit the toilet during the lessons, but this adversely affected the psychosomatic development of the maturing girls and led to their social isolation.

In N-KSM, the bladder is deprived of the protective layer of GAGs, so the superficial layer becomes susceptible to different factors, for example, bacteria, viruses, inflammatory factors, and irritants (urine pH).

The pathogenesis of metaplasia of the urinary bladder is still not fully known. Many studies in this regard have focused on endocrine factors and related hormonal disorders in menopausal women [24]. These studies did not involve children or pubescent girls. Because of conventional treatment modalities, for example, anti-inflammatory therapy, vitamin A supplementation, cystoclysis with a gentamycin solution, we are compelled to look for alternative therapies for effectively treating children and adults with bladder mucosa metaplasia.

Hormonal disorders leading to reduced levels of progesterone cannot be considered the causes of metaplasia because metaplasia also occurs in boys and nonmenstruating girls.

Chronic inflammatory changes in the submucosal layer of the urinary bladder can lead to metaplasia-like changes. Inflammatory infiltration observed on histopathological examination and the findings of other clinical tests highlight the fact that existing disorders lead to an imbalance between the action of proinflammatory factors and immunoregulatory abilities of the immune system [25]. Current views on the role of the immune system consider its defensive, tolerogenic, and progenerative functions as basic elements of the hemostasis system. The role of the immune system in this disorder depends mainly on the thymus-dependent lymphocytes, which play critical immunoregulatory functions. Hemostasis in an organism depends on the right functioning and coordination between the 3 integral systems in the organism, that is, the nervous system, endocrine system, and immune system. Therefore, future studies should focus on immunological factors to determine whether treating defects in the immune system is indicated or contraindicated in patients with metaplasia of the urinary bladder.

The symptoms of metaplasia are typical: pollakiuria, feeling of fullness in the bladder, difficulties in initiating micturition, pain on micturition, and urinary urgencies. Changes in bladder activity can also be determined through a medical interview. Maturing girls who complain of nontypical symptoms related to micturition and urinary tract infection should be referred for diagnostic cystoscopy. These painful complaints mostly occur in the day time, disturbing normal functioning.

## 5. Conclusions

Metaplasia of the urinary bladder occurs in children and adolescents. Symptomatic treatment is aimed to improve the patients' quality of life and disease prognosis. However, further studies are required on the etiology and pathogenesis of changes involved in this disease to establish an effective treatment modality. Children who are psychologically less stable are not able to deal with dysuric disorders; therefore, these children would require multispecialty management to enable them to function to their full potential in society.
